# Morality and disgust: insights from obsessive compulsive disorder

**DOI:** 10.3389/fpsyt.2012.00113

**Published:** 2013-01-03

**Authors:** Carmelo M. Vicario

**Affiliations:** ^1^School of Psychology, University of QueenslandBrisbane, QLD, Australia; ^2^Faculty of Motor Science, University of PalermoPalermo, Italy

**A commentary on**

**Neural correlates of moral sensitivity in obsessive compulsive disorder**

*by Harrison, B. J., Pujol, J., Soriano-Mas, C., Hernández-Ribas, R., López-Solà, M., Ortiz, H., et al. (2012). Arch. Gen. Psychiatry 69, 741–749*.

In a recent issue of *Archives of General Psychiatry*, Harrison et al. (2012) provided the first evidence of neural correlates on moral sensitivity in Obsessive Compulsive Disorder (OCD), which is believed to be heightened in this clinical population. This study aimed to explore whether subjects with OCD showed increased ventromedial prefrontal and orbitofrontal cortex responses in a functional magnetic resonance imaging study of difficult moral decision-making.

These brain areas were found to be active on healthy humans experiencing moral emotions such as indignation or moral disgust (see Moll et al., 2005 for a complete review).

The results are striking in so far as they show that patients with OCD demonstrated significantly increased activation of the ventral frontal cortex, particularly of the medial orbitofrontal cortex. Moreover, significant positive associations were documented between the patients' DY-BOCS ratings of total symptom severity and the activation of the anterior insula (Harrison et al., 2012).

The evidence from this study offers the opportunity to discuss two emerging related issues.

First, it adds further support on a clinical model to the view that visceral and moral disgust share, at least in part, common neural and cognitive mechanisms (Jones, 2007). In fact, OCD is also characterized by an altered representation of disgust at sensory (Tsao and McKay, 2004) and affective (Shapira et al., 2003) level.

Thus, their heightened moral sensitivity might reflect an enhanced disgust for immoral outcomes.

This hypothesis is supported by previous evidence coming from neuroimaging studies. For instance, an overlapped recruitment of ventromedial prefrontal and orbitofrontal cortices have already been reported during the processing of both sensorial and moral disgust (Moll et al., 2005).

A similar result was announced for the insula. For example, Wicker et al. (2003) have discovered that the anterior insula was activated both during the observation of disgusted facial expressions and during the feeling of disgust evoked by unpleasant odors. Moreover, Sanfey et al. (2003) found an activation of this region in healthy subjects who received unfair monetary offers, which are known to elicit moral disgust.

All these findings suggest that, the heightened moral sensitivity in OCD can be grounded on the same neural mechanisms responsible for their altered sensitivity to outcomes which can induce sensory and emotional disgust (Shapira et al., 2003; Tsao and McKay, 2004).

Another remarkable aspect deserving some discussion is that OCD participants were not different from healthy control subjects in the subjective rating of the moral dilemmas, despite the difference reported in neural activation. This result, which probably argues some distinction between the patients' increased neural responses and their perceived emotional experience during a moral dilemma (Harrison et al., 2012), can be discussed calling into question the interoceptive awareness which might be deranged in this clinical population.

This suggestion is supported by the recent evidence of a negative correlation between interoception and anxiety (Pollatos et al., 2009), which is known to affects OCD. Moreover, Zaki et al. (2012) have recently found a common cluster of activation for interoception and emotional experience in the anterior insula, a region clearly deranged in this clinical population (Song et al., 2011).
